# Supplementary figures and images for: Network Events on Multiple Space and Time Scales in Cultured Neural Networks and in a Stochastic Rate Model
Source: PLoS Comput Biol. 2015 Nov 11;11(11):e1004547. doi: 10.1371/journal.pcbi.1004547 (PMC4641680; doi:10.1371/journal.pcbi.1004547)

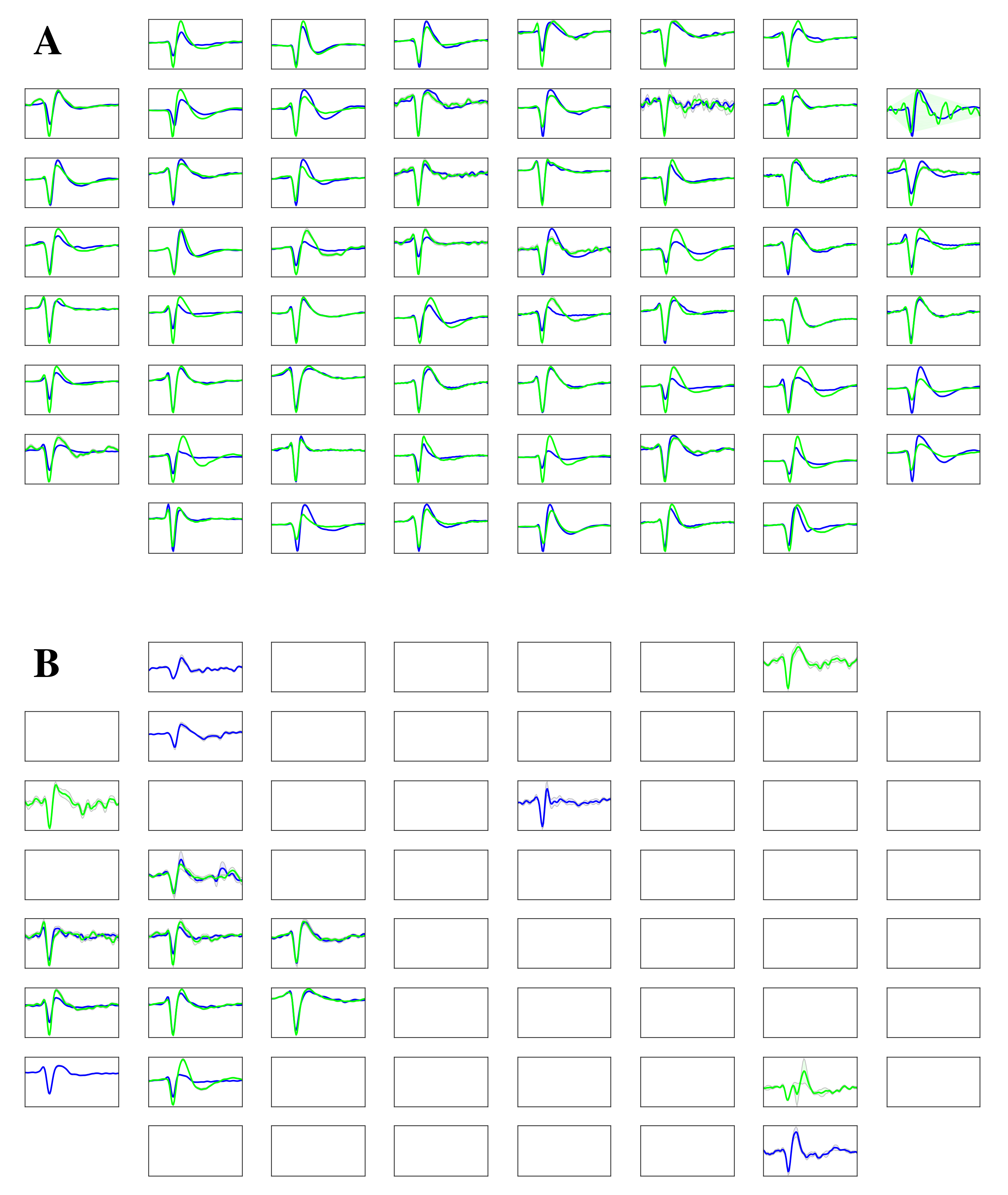

Supplement: S1 Fig — Waveforms of the recorded spikes across all MEA electrodes, comparing the earliest and latest used recordings (about 40 minutes each, separated by about 34 hours). In most cases the waveforms for the two recordings are remarkably similar, and when they are not, no systematic trend in the differences is observed. Panel A: average wave-form for each electrode; blue lines refer to the earliest recording, green line to the latest. For each electrode, we averaged only the first spike detected (if any) for that electrode in each network spike, to best isolate local excitability properties from global network effects during the development of the network spike. The shaded colored strips are ±standard deviation. Panel B: waveforms averaged under a stricter selection: only the very first spike of the entire network in each network spike is considered, which clearly selects fewer leading electrodes (only electrodes with 5 or more recorded first spikes are shown). (TIF) [file pcbi.1004547.s001.tif]

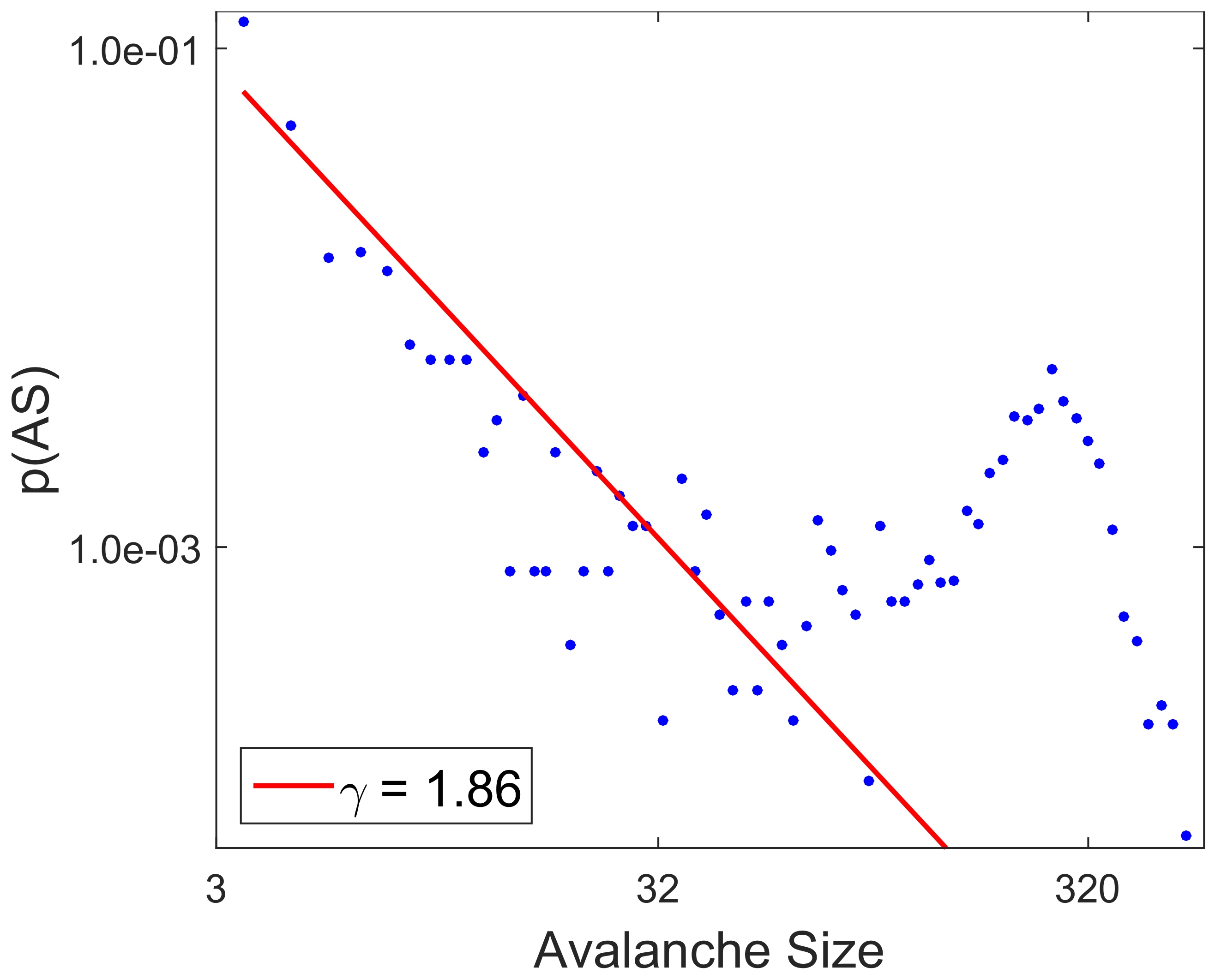

Supplement: S2 Fig — The distribution is sparser but qualitatively compatible with the distribution in Fig 6, panel F; in particular, the distribution shows a prominent peak for high-size avalanches, consistently with the interpretation, given in connection with Fig 5, of high excitability. (TIF) [file pcbi.1004547.s002.tif]

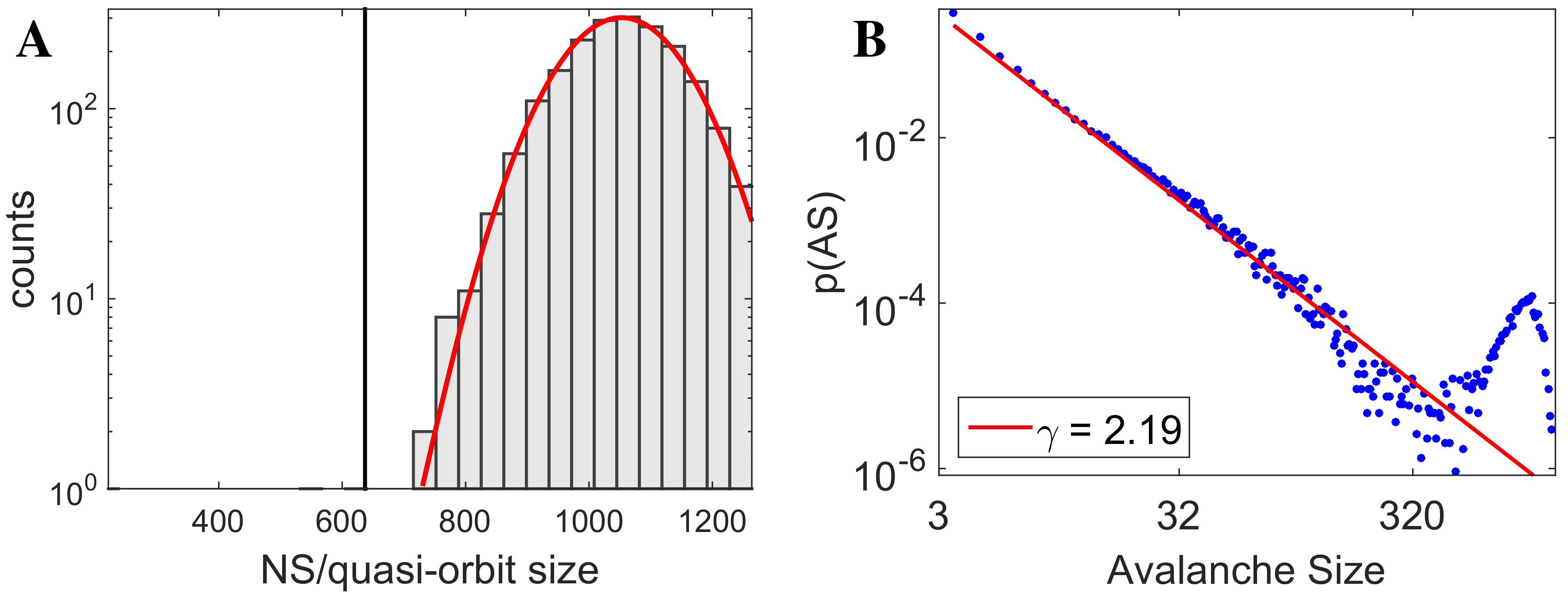

Supplement: S3 Fig — Data from a simulation of the network corresponding to the point (wexc, winh) = (1.2, 0.4), on the right of the Hopf bifurcation of Fig 2. Panel A: NS-quasi orbits size distribution; the high excitability of the network generates increasingly stereotyped network spikes, whilst the contribution from noise-induced quasi-orbits vanishes. Panel B: the distribution of avalanche sizes, while preserving the small-size power-law tail, is increasingly dominated by the high-size bump associated with network spikes. See also Figs 5 and 6. (TIF) [file pcbi.1004547.s003.tif]
